# New Insights Into Heat Shock Protein 90 in the Pathogenesis of Pulmonary Arterial Hypertension

**DOI:** 10.3389/fphys.2020.01081

**Published:** 2020-09-15

**Authors:** Liqing Hu, Rui Zhao, Qinglian Liu, Qianbin Li

**Affiliations:** ^1^Department of Medicinal Chemistry, Xiangya School of Pharmaceutical Sciences, Central South University, Changsha, China; ^2^Department of Physiology and Biophysics, School of Medicine, Virginia Commonwealth University, Richmond, VA, United States; ^3^The First Clinical School, Shandong University of Traditional Chinese Medicine, Jinan, China

**Keywords:** pulmonary arterial hypertension, heat shock protein 90, soluble guanylate cyclase, AMP-activated protein kinase, pathogenesis, novel therapeutic options

## Abstract

Pulmonary arterial hypertension (PAH) is a multifactorial and progressive disorder. This disease is characterized by vasoconstriction and vascular remodeling, which results in increased pulmonary artery pressure and pulmonary vascular resistance. Although extensive studies have been carried out to understand the etiology, it is still unclear what intracellular factors contribute and integrate these pathological features. Heat shock protein 90 (Hsp90), a ubiquitous and essential molecular chaperone, is involved in the maturation of many proteins. An increasing number of studies have revealed direct connections between abnormal Hsp90 expression and cellular factors related to PAH, such as soluble guanylate cyclase and AMP-activated protein kinase. These studies suggest that the Hsp90 regulatory network is a major predictor of poor outcomes, providing novel insights into the pathogenesis of PAH. For the first time, this review summarizes the interplay between the Hsp90 dysregulation and different proteins involved in PAH development, shedding novel insights into the intrinsic pathogenesis and potentially novel therapeutic strategies for this devastating disease.

## Introduction

Pulmonary arterial hypertension (PAH) is a life-threatening condition characterized by high blood pressure in the arteries that flow from the heart to the lung. Different from systemic hypertension, a decreased compliance of the pulmonary arterial system and progressive narrowing of the pulmonary arteries are the key features of PAH. These features are mainly caused by vasoconstriction and vascular remodeling, which eventually lead to high right ventricular (RV) afterload, RV failure, and ultimately death (Tuder et al., 2013).

PAH is defined by a mean pulmonary artery pressure (PAP) > 20 mmHg at rest, a normal capillary wedge pressure ≤ 15 mmHg, and a pulmonary vascular resistance ≥ 3 Wood units (Simonneau et al., 2019). The prevalence and geographic distribution of PAH vary based on disease type and etiology. Although worldwide prevalence remains unclear, the estimated incidence of PAH normally ranges from 2.0 to 7.6 cases per million adults per year, and its prevalence varies from 11 to 26 cases per million (Thenappan et al., 2018). To date, PAH still remains relatively incurable despite the significant signs of progress in the disease awareness, development of diagnostics, and therapeutics. Moreover, this debilitating disease is accompanied by high morbidity with a 1-year mortality rate of 15–20% and a poor median survival of 7 years (Benza et al., 2010; Thenappan et al., 2010). Hence, it is paramount to find efficient PAH therapies to improve long-term outcomes for PAH patients.

The pathogenesis of PAH is complicated. The traditional view supports that elevated PAP is caused by the imbalance of endogenous vascular factors such as nitric oxide (NO), prostacyclin, endothelin, and thromboxane (Budhiraja et al., 2004; Dunham-Snary et al., 2017). Clinically, high PAP is the primary symptom to be dealt with, which is the major strategy for current therapy, i.e., diuretics, calcium channel blockers, anticoagulants, inhaled NO donors, and targeted therapy including soluble guanylate cyclase (sGC) stimulators, phosphodiesterase-5 inhibitors, endothelin receptor antagonists, and prostacyclin analogs. However, all these strategies, mainly focusing on vascular relaxation with little emphasis on vascular remodeling, are gradually running into drawbacks and bottlenecks such as drug resistance, systematic hypotension, and unsatisfactory long-term use, although they could improve patient’s functional capacity and hemodynamics when used alone or in combination (Galie et al., 2016). Growing evidence supports that vascular remodeling plays an important role in the pathogenesis of PAH (Humbert et al., 2004; Rabinovitch, 2012). Recent studies have highlighted the mechanisms on regulating proliferative vascular remodeling and thus advanced our understanding of PAH pathogenesis from novel genetic and epigenetic factors to cell metabolism and DNA damage (Thompson and Lawrie, 2017). Vascular remodeling is mainly characterized as abnormal proliferation and migration of pulmonary artery smooth muscle cells (PASMCs). These abnormal phenotypes in pulmonary vascular cells under PAH result in vasoconstriction and loss of elasticity of vascular wall, which eventually leads to increased PAP, RV hypertrophy, and RV overload. Accordingly, many attempts and efforts to treat PAH through inhibiting vascular remodeling have been performed (Dai Z. et al., 2018; Dai and Zhao, 2019). Accumulating evidence suggests that bromodomain and extra-terminal motif (BET) is implicated in the pathogenesis of PAH. More promisingly, RVX208, a clinically available BET inhibitor, has been shown to improve hemodynamics and reverse pulmonary vascular remodeling without attenuation of RV hypertrophy or mortality in animals with PAH (Dai and Zhao, 2019). Furthermore, previous studies have shown that hypoxia-inducible factor-2α signaling is activated in lung tissues from patients with PAH and distinct rodent PAH models. More importantly, pharmacological inhibition of hypoxia-inducible factor-2α has been shown to reduce obliterative pulmonary vascular remodeling in hypoxia-exposed rats (Dai Z. et al., 2018). However, in other studies, inhibition of vascular remodeling alone is not able to rapidly decrease blood pressure, the primary and acute symptom of PAH, which in turn promotes a change of vascular status and leads to a failure of PAH treatment (Shimoda and Laurie, 2013; Liu et al., 2018; Shi et al., 2018). In this regard, heat shock protein 90 (Hsp90) chaperone machinery has emerged as a promising axis that can simultaneously regulate the expression of multiple aberrant proteins implicated in PAH development and progression (Taipale et al., 2010; Paulin et al., 2011).

## Heat Shock Protein 90

Heat shock proteins (HSPs are a collection of conserved families of proteins that are induced by various cellular and environmental stresses such as high temperature, hypoxic damage, and oxidative stress (Young et al., 2004). Traditionally, many HSPs have also been known as molecular chaperones due to their essential roles in processes involved in maintaining cellular protein homeostasis, including facilitating protein folding and transportation and maintaining mature structures and functions of proteins (Javid et al., 2007). Hsp90, a unique family of HSPs with a molecular weight of 90 kDa, is a class of evolutionarily conserved and abundant molecular chaperones that mediate many fundamental cellular processes (Zhao et al., 2005; Schopf et al., 2017). In general, Hsp90 accounts for 1–2% of the total cellular protein under non-stressful conditions, and its level rises up to 4–6% in response to stressful conditions (Taipale et al., 2010; Finka and Goloubinoff, 2013). So far, four isoforms have been discovered for Hsp90 in humans (Sreedhar et al., 2004): Hsp90α, Hsp90β, glucose-regulated protein 94, and tumor necrosis factor receptor-associated protein 1. Both Hsp90α and Hsp90β reside in the cytosol. However, the expression of Hsp90α is induced upon cellular stresses, whereas Hsp90β is constitutively expressed. GRP94 is located in the endoplasmic reticulum, and tumor necrosis factor receptor-associated protein 1 is present in the mitochondria.

### Structure of Heat Shock Protein 90

**FIGURE 1:**
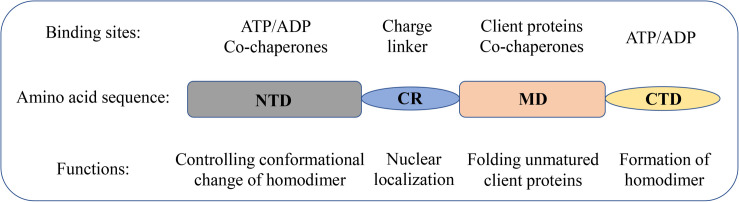
Domain structure of Hsp90 family members in humans. Schematic representation of the domain structure of Hsp90 isoforms together with the biological functions of each domain.

The structural and molecular characteristics of Hsp90 have been systematically reviewed elsewhere (Li and Buchner, 2013; Hoter et al., 2018; Li et al., 2020). Only a brief description is provided in this review. Hsp90 exists as a homodimer. The monomer comprises of four domains (Figure 1): an N-terminal dimerization domain (NTD), a charged region (CR) of a variable length, a middle domain (MD), and a C-terminal domain (CTD) (Ali et al., 2006; Pullen and Bolon, 2011; Street et al., 2011). The four domains are flexibly linked. The domain organization is conserved from bacteria to humans except for the CR domain, which is only present in eukaryotic Hsp90. The NTD binds adenosine triphosphate (ATP). Interestingly, several conserved residues of the ATP-binding site in NTD form a “lid” that closes over the nucleotide-binding pocket in the ATP-bound state but is open in the ADP-bound state (Ali et al., 2006). These residues are essential for the ATPase activity of Hsp90, which is indispensable for the chaperone cycle and binding client proteins. The CR domain is highly charged and has a variable length and amino acid composition, suggesting increased flexibility and dynamics to cope with the crowded environment in eukaryotic cells (Shiau et al., 2006; Tsutsumi et al., 2009). The MD of Hsp90 contains crucial catalytic residues for forming the composite ATPase site, which interacts with the γ-phosphate of ATP and thus promotes ATP hydrolysis (Meyer et al., 2003; Huai et al., 2005). Moreover, the MD contributes to the interaction sites for client proteins and some co-chaperones (Meyer et al., 2003, 2004; Huai et al., 2005). For instance, the co-chaperone Aha1 binds with MD to modulate the active conformation of the catalytic loop, which consequently stimulates the ATPase activity of Hsp90 (Meyer et al., 2004). The CTD is responsible for the inherent dimerization. Moreover, following the CTD is a highly conserved pentapeptide, MEEVD, which serves as the docking site for the interaction with co-chaperones containing tetratricopeptide repeat clamp (Scheufler et al., 2000; Ratzke et al., 2010).

### Conformation Dynamics of Heat Shock Protein 90

**FIGURE 2:**
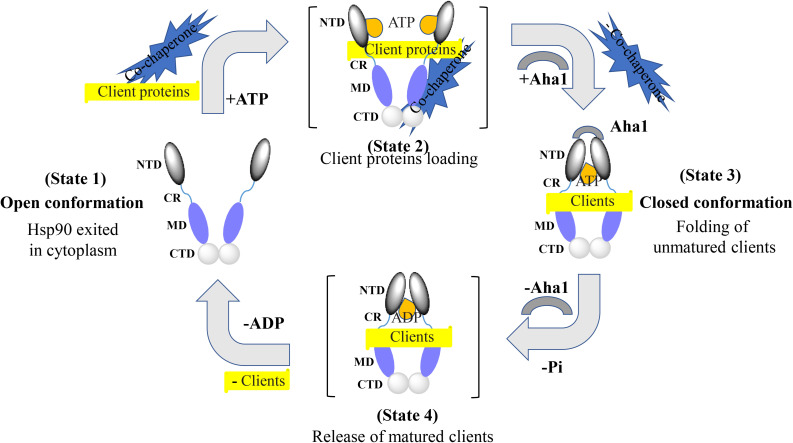
Hsp90 chaperone cycle. Open conformation state is likely to be the most efficient at binding client proteins. Addition of ATP altering the relationship between the NTD and MD, resulting in a transient NTD/MD conformation. Co-chaperones assist in loading client proteins onto Hsp90. Aha1 increases Hsp90 ATPase activity, contributing to the closing of the NTD. Client proteins would remain bound due to the hydrophobic surfaces still present on the MD and CTD. Client proteins are folded during the process of NTD closure. Finally, nucleotide hydrolysis results in the very compact ADP state and the release of client proteins. Open conformation of the Hsp90 NTD is restored for the next chaperone cycle.

Extensive structural studies revealed that the Hsp90 chaperone cycle could be divided into distinct conformations, which seem to be in a dynamic equilibrium (Shiau et al., 2006; Mickler et al., 2009). In the apo state (Figure 2), Hsp90 adopts an open V-shaped form predominantly, termed “open conformation” (Graf et al., 2009). Then, binding and hydrolysis of ATP drives the conformational changes and leads to the formation of the first intermediate state, in which the ATP lid is closed, but the NTD are still open (Hessling et al., 2009). The unfolded substrates are recognized by co-chaperones and loaded onto Hsp90 during this process (Genest et al., 2019). Subsequently, Hsp90 ATPase activity is triggered by co-chaperone Aha1 along with the dissociation of other co-chaperones such as Hsp70 and Hop. This promotes the closure of the Hsp90 homodimer. Now, Hsp90 is in the “closed conformation” (Li et al., 2013). Then, the dimerization leads to the formation of the second intermediate state, in which the MD repositions and interacts with the NTD. The ATP hydrolysis is catalyzed in this fully closed state, resulting in substrate folding. The following opening of the NTD of the Hsp90 dimer restores the initial “open conformation” (Shiau et al., 2006; Hessling et al., 2009).

### Functions of Heat Shock Protein 90

In cells, Hsp90 is involved in diverse cellular processes, including protein folding, maturation, post-translational modification, recognition, degradation, signaling transduction, cell cycle, and cellular differentiation (Taipale et al., 2010; Hoter et al., 2018). Several published articles have extensively reviewed the roles of Hsp90 in protein folding, maturation, and preventing protein aggregation (Shiau et al., 2006; Taipale et al., 2010; Kirschke et al., 2014; Genest et al., 2019). Recently, the post-translational modifications of Hsp90 have gained an increasing significance. These modifications include phosphorylation, acetylation, nitrosylation, and methylation. Interestingly, these covalent modifications influence the chaperone activity of Hsp90 and, thus, the maturation of client proteins (Mollapour and Neckers, 2012). One widely accepted hypothesis on the recognition of Hsp90 for its clients is that Hsp90 recognizes certain conformations or the stability of selected clients rather than its primary sequence (Falsone et al., 2004; Muller et al., 2005). However, further studies still need to resolve this conundrum in the future. Several reports have shown that Hsp90 is also required for facilitating protein degradation through involvement in the ubiquitin–proteasome pathway mediated by the carboxyl terminus of Hsp70-interacting protein (Goasduff and Cederbaum, 2000; Fan et al., 2005; Xia et al., 2007). The clients of Hsp90 have been shown to associate with a large number of signaling pathways, such as protein kinases and steroid hormone receptors (Sato et al., 2000; Kazlauskas et al., 2001; Theodoraki and Caplan, 2012; Boczek et al., 2015). Hence, Hsp90 seems to be essential in the maturation and protection of the protein functions as a key chaperone implicated in cell signaling. Many of the client proteins chaperoned by Hsp90 are essential for the progression of various diseases, including cancer, Alzheimer’s disease, and other neurodegenerative diseases, as well as viral and bacterial infections (Zuehlke et al., 2018; Li et al., 2020). Hence, it has been proposed that targeting Hsp90 is an effective way of combating a large range of diseases (Den and Lu, 2012). Interestingly, the functions of Hsp90 isoforms were also dramatically affected by the stages of the cell cycle and differentiation, which differ in the level of cellular ATP (Nguyen et al., 2013; Echeverria et al., 2016). As mentioned earlier, ATP binds to the NTD of Hsp90, and the hydrolysis of ATP provides energy for its functions. Therefore, competitively inhibiting the ATP-binding process with Hsp90 inhibitors has gained significant attention in disease treatment, and several Hsp90 inhibitors have entered clinical trials currently (Li et al., 2020).

### Inhibitors of Heat Shock Protein 90

**FIGURE 3:**
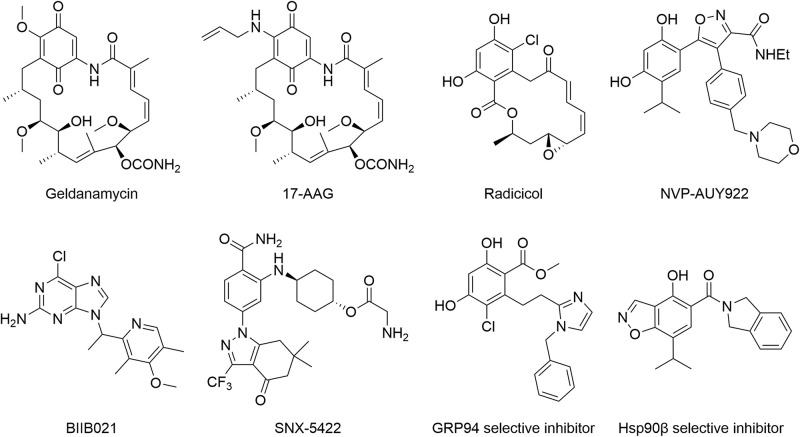
Structures of Hsp90 inhibitors.

In the past two decades, Hsp90 has received tremendous attention from medicinal chemists, and thus, extensive efforts have been made in developing various Hsp90 inhibitors (Figure 3) via different experimental approaches (Xiao and Liu, 2020). The first generation of Hsp90 inhibitors is natural products such as geldanamycin, radicicol, and their derivatives. 17-AAG was derived from geldanamycin. It was the first Hsp90 inhibitor to enter the clinical trial in 1999. NVP-AUY922 was the first resorcinol based small-molecule Hsp90 inhibitor through high-throughput screening and structure-based optimization. BIIB021, a purine analog identified from the Hsp90 and ATP cocrystal structure studies, was the first non-geldanamycin small-molecule Hsp90 inhibitor to enter the clinical evaluation. SNX-5422 bearing a benzamide scaffold was identified by a chemo-proteomics based screening approach and entered the clinical trial in 2008. Unfortunately, none of these inhibitors has been approved up to date, although many have been tested in clinical trials (Xiao and Liu, 2020). The lack of drug-like properties, organ toxicity and/or drug resistance are the main obstacles for preventing these Hsp90 inhibitors from reaching the market. Hence, improving drug selectivity is considered as a practical strategy for minimizing drug toxicity. GRP94-selective inhibitors were first developed based on the lowest similarity with the other three Hsp90 isoforms. Given the cocrystal structures of radicicol bound to Hsp90α and Hsp90β and two different amino acid residues at the ATP binding sites, a series of Hsp90β selectively targeted inhibitors were developed. Up to now, Hsp90 inhibitors in either clinical or preclinical evaluations have been mainly developed for cancer therapy, however, none has reached the market. In this context, new indications, as well as new chemical structures, should be considered during the development of novel Hsp90 inhibitors.

## Heat Shock Protein 90 in Pulmonary Arterial Hypertension

As a ubiquitous chaperone, Hsp90 has received much attention due to its important roles in cancer biology, regulating proliferation, growth, differentiation, adhesion, invasion, metastasis, angiogenesis, and apoptosis. Thus, Hsp90 is probably one of the best-studied HSP proteins (Wu et al., 2017). Recently, several reports suggested the involvement of Hsp90 in the pathogenesis of PAH, especially in vascular remodeling, although the precise pathogenesis mechanisms of PAH remain to be elucidated (Sakao and Tatsumi, 2011; Wang et al., 2016; Boucherat et al., 2017, 2018). Studies have shown that the level of Hsp90 was increased in both plasma and membrane walls of pulmonary arterioles from PAH patients (Wang et al., 2016). Moreover, Hsp90 inhibitor 17-AAG has been shown to improve pulmonary vascular remodeling via suppressing the excessive proliferation and migration of PASMCs (Wang et al., 2016). Notably, findings indicate that the accumulation of Hsp90 in PASMC mitochondria was a hallmark of PAH development and a key regulator of mitochondrial homeostasis contributing to vascular remodeling in PAH (Boucherat et al., 2018). Not surprisingly, cytosolic Hsp90 stimulates PASMC proliferation by stabilizing key signaling proteins involved in PAH development and progression. Moreover, study results demonstrated that the mitochondrial accumulation of Hsp90 in PASMCs of PAH contributes to their proliferation and survival under environmental stresses and thus promotes vascular remodeling (Boucherat et al., 2018). Herein, accumulation of Hsp90 in mitochondria represents a feature in PAH vascular remodeling and thus may be a weakness to exploit. Gamitrinib, a small molecule designed to target selectively Hsp90 in mitochondria, was associated with antiproliferation activity in preclinical models with no overt organ or systemic toxicity (Kang et al., 2009). It was demonstrated that targeted inhibition of mitochondrial Hsp90 with Gamitrinib reversed pulmonary vascular remodeling and improved cardiac output in two PAH models without noticeable toxicity (Boucherat et al., 2018). Thus, pharmacological inhibition of Hsp90 is a promising avenue to improve the clinical outcomes of patients with PAH, and drugs that target Hsp90 in mitochondria will show more advantages in PAH treatment (Boucherat et al., 2018). However, the specific mechanisms of Hsp90 in PAH pathogenesis still remain to be illuminated, although it is clearly relevant to PAH development. Unexpectedly, Hsp90 is implicated in regulating the function of many proteins essential for the PAH development and progression, such as sGC and AMP-activated protein kinase (AMPK).

### Heat Shock Protein 90 and Soluble Guanylate Cyclase

sGC, as the best established NO physiological receptor, is a heterodimer made up of similar α and β subunits (Derbyshire and Marletta, 2012; Childers and Garcin, 2018; Kang et al., 2019). The binding of NO to sGC heme prosthetic group results in the synthesis of the second messenger cyclic guanosine monophosphate, establishing the NO–sGC– cyclic guanosine monophosphate signaling pathway, which is essential for maintaining cardiovascular health (Stasch et al., 2011). Accordingly, substantial experimental data have suggested the impaired sGC activity in the pathogenesis of PAH (Schermuly et al., 2008; Ghofrani and Grimminger, 2009; Stasch et al., 2011; Dasgupta et al., 2015). Interestingly, sGC expression, especially sGC β1, was upregulated compared with control subjects in the PAH patients’ pulmonary arterial tissue samples as well as experimental animal models of PAH (Schermuly et al., 2008).

**FIGURE 4:**
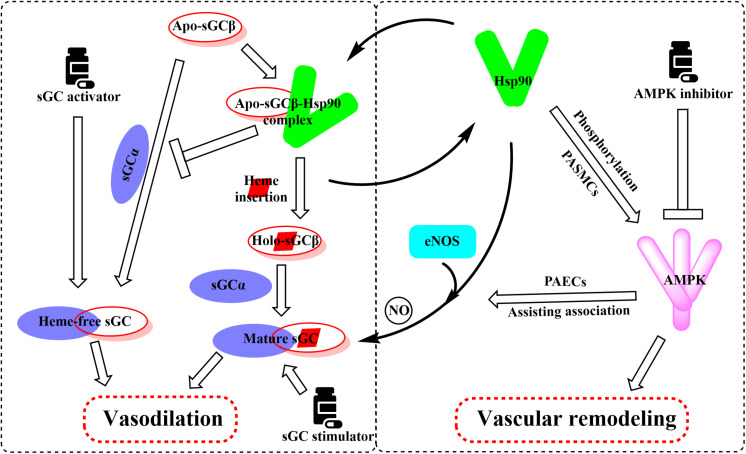
Overview of the important roles of Hsp90 in the regulation of sGC and AMPK involved in the pathogenesis of PAH. Hsp90 drives heme insertion into apo-sGCβ after the complex of Hsp90 and apo-sGCβ formation. Meanwhile, Hsp90 also prevents apo-sGCβ’s premature interaction with sGCα to form a heme-free, non-functional sGC heterodimer. Hsp90 dissociates from sGCβ once heme insertion is complete, allowing holo-sGCβ to partner with sGCα and to form a mature and functional sGC heterodimer. AMPK is a client of Hsp90, and Hsp90 interferes with the function of the AMPK complex by mediating the phosphorylation of AMPK in PASMCs. Activation of AMPK in PAECs assists the complex formation between eNOS and Hsp90, promoting eNOS-mediated NO production.

Hsp90 was reported to associate with several heme proteins and influence their functions, stabilization, maturation, and activities (McClellan et al., 2007). In 2003, Hsp90 was first reported to associate with sGC. Subsequent studies identified the effects of Hsp90 on sGC-mediated vascular relaxation by regulating the stability of sGC (Yetik-Anacak et al., 2006; Nedvetsky et al., 2008). Remarkably, a myriad of works on how Hsp90 regulates sGC function has been carried out in the last decade, and the related research progress was also summarized (Ghosh and Stuehr, 2017). Therefore, we mainly present a brief overview to provide their basic results for understanding the Hsp90 impact on sGC under physiological and pathological conditions. Hsp90 regulates sGC, involving the association of Hsp90 MD with two regions of sGC, by a dual mechanism (Papapetropoulos et al., 2005; Dai et al., 2019). On the one hand, Hsp90 drives heme insertion into apo-sGC β1 after forming a complex. Once heme insertion is complete, Hsp90 dissociates from sGC β1, which partners with sGC α1 to form a mature and functional sGC heterodimer. On the other hand, apo-sGC β1 associates with Hsp90 much more strongly than sGC α1, which prevents the formation of heme-free and non-functional sGC heterodimer (Dai et al., 2019). It is well established that oxidative stress, which plays a major role in the development of pulmonary vascular remodeling and a consequent increase of pulmonary pressure, can cause the oxidation of the sGC heme resulting in desensitization to NO signaling (Shah et al., 2018). NO stimulates the insertion of heme in apo-sGC-β1, the dissociation of Hsp90 from the complex, and the association of sGC-α1 to form the active enzyme (Ghosh et al., 2014). It seems that impaired NO and the upregulation of Hsp90 in PAH break the insertion of heme into sGC-β1 and the equilibrium between apo-sGC-β1 and holo-sGC-β1 (Ghosh et al., 2014; Dai et al., 2019). However, how this equilibrium is managed remains to be elucidated. In this way, the activity of sGC heterodimer may be impaired despite the elevated sGC level under pathophysiology of PAH. Given that dysregulation of the sGC signaling pathway has been associated with PAH and cardiovascular disease, several stimulators and activators of sGC have been developed as therapeutics. The heme-dependent sGC stimulators and the heme-independent sGC activators have distinct mechanisms of action (Follmann et al., 2013). sGC stimulators share a dual mode of action: they synergize with endogenous NO, and furthermore, they are also capable of directly stimulating the mature sGC in a manner that is independent from NO. In contrast, sGC activators increase enzymatic activity of the oxidized heme or heme-deficient apo-sGC without relying on NO. The pharmacologic sGC activators were found to mimic all the changes in sGC β1 protein associations and consequent increase in the sGC heterodimer formation (Dai et al., 2019). Thus, inhibition of Hsp90 may further enhance the efficacy of sGC activators to treat PAH by increasing the levels of heme-free sGC (Figure 4).

### Heat Shock Protein 90 and AMP-Activated Protein Kinase

AMPK is a heterotrimeric complex consisting of a catalytic subunit α (α1 and α2) and two regulatory subunits β and γ and primarily regulates the energy homeostasis in eukaryotes (Ross et al., 2016). AMPK activity requires phosphorylation of Thr172 in the activation loop of the kinase domain. In a cellular context, liver kinase B1 and calcium/calmodulin-dependent protein kinase β have been identified as the two major upstream kinases capable of phosphorylating Thr172, which is critical for significant activation of AMPK (Willows et al., 2017). As a nutrient sensor, AMPK is critical for lung function and involved in many lung diseases, especially PAH (Ibe et al., 2013). Recent studies suggest that inhibition of AMPK prevents the development of PAH via decreasing vascular remodeling characterized by abnormal PASMC proliferation and migration (Dai J. et al., 2018). The concentrations of phosphorylated AMPK in PASMCs of PAH and hypoxic mouse PASMCs appeared to be elevated. Moreover, compound C, an AMPK inhibitor, prevents hypoxia-induced PAH *in vivo* (Ibe et al., 2013). Also, this study suggests that AMPKs α1 and α2 play differential roles in the survival of PASMCs during hypoxia (Ibe et al., 2013). The activation of α1 prevents apoptosis, whereas the activation of α2 stimulates autophagy, both of the effects promoting PASMC abnormal proliferation. These results show that inhibition of AMPK phosphorylation presents a possible therapeutic strategy in the treatment of PAH.

Previous reports have shown that Hsp90 selectively interacts with and stabilizes key signaling proteins to regulate cell survival and proliferation via preferentially interfering with their metabolic balance (Schopf et al., 2017). Accordingly, several investigators have focused on the interaction between Hsp90 and AMPK, and results demonstrate that Hsp90 chaperones regulate cell metabolism via mediating the activation of AMPK (Zhang et al., 2012). Furthermore, Hsp90 interacts with AMPK and maintains its kinase activity, which in turn is required for the phosphorylation of AMPK and its substrate, acetyl-CoA carboxylase (Zhang et al., 2012). Hsp90 inhibitors reduced the enzymatic activity of AMPK by inhibiting the complex formed by Hsp90 with AMPK. Moreover, dissociation of the γ subunit from AMPK triggered by Hsp90 inhibitor weakens the stability of the heterotrimeric complex, as the direct Hsp90 inhibition disassembles the AMPK-Hsp90 complex and liberates the α subunit from the AMPK complex, resulting in the decline of enzymatic activity and phosphorylation of acetyl-CoA carboxylase (Zhang et al., 2012). Therefore, the effects of Hsp90 inhibition in PAH therapy are likely to depress the AMPK signaling pathway (Figure 4).

As previously mentioned, the impairment of NO production has long been considered to be associated with the pathogenesis of PAH (Ghofrani and Grimminger, 2009; Stasch et al., 2011). Validated data indicate that endothelial NO synthase (eNOS) generates NO as a homodimer in the presence of heme. Hsp90 associates with eNOS to form a complex, which stimulates the production of NO (García-Cardeña et al., 1998). Previous studies demonstrate that Hsp90 plays an important role in the maintenance of eNOS dimer structure and function (Chen et al., 2014). Moreover, inhibition of Hsp90 or silencing of Hsp90 causes the eNOS dimer to dissociate into monomers and exposes Ser1179 and Thr497 on the monomers to various phosphatases, resulting in eNOS dephosphorylation and degradation. Structure studies showed that Hsp90 associates with the oxygenase domain of eNOS in which eNOS also associates with many kinds of different cofactors or substrates, such as Heme, tetrahydrobiopterin (BH4), and L-arginine (Balligand, 2002; Chen et al., 2017). In fact, Hsp90 uniquely regulates eNOS through specifically changing a few eNOS cofactor affinity, such as reduced nicotinamide adenine nucleotide phosphate and calcium (Ca^2+^)/calmodulin (Chen et al., 2017). Meanwhile, sufficient study data confirm that mitochondrial dysfunction in pulmonary arterial endothelial cells (PAECs) decreases the cellular ATP levels and Hsp90 chaperone activity, resulting in a reduced interaction between Hsp90 and eNOS in cardiovascular disorders (Sud et al., 2008). Hence, the eNOS activities and NO generation were decreased despite the overall elevated Hsp90 level in PAH. Interestingly, some research results suggest that AMPK activity is essential for eNOS activation via promoting the association between eNOS and Hsp90 (Schulz et al., 2005; Figure 4). In contrast, pharmacological or molecular inhibition of AMPK did not alter the level of eNOS phosphorylation (Schulz et al., 2005; Chen et al., 2009). Also, clinically relevant concentrations of metformin, as a most commonly used AMPK activator, activate eNOS and NO bioactivity in an AMPK-dependent manner (Davis et al., 2006). Taken together, although divergent findings exist, Hsp90 may play a significant role in PAH by interacting with AMPK and eNOS pathways. More research is needed to understand the effects of Hsp90 in PAH development.

Various studies have shown that overexpression of Hsp90 was related to many diseases such as cancer and chronic degenerative diseases (Richardson et al., 2011; Fuhrmann-Stroissnigg et al., 2017). However, the studies that focused on the relationships between Hsp90 accumulation and PAH development are limited, and the underlying roles of Hsp90 in the occurrence and progression of PAH remain unclear. Our review is the first to propose the possible mechanisms of Hsp90 in the pathogenesis of PAH by comprehensively summarizing the interactions of Hsp90 with sGC and AMPK signaling pathways (Figure 4).

## Prospective of Heat Shock Protein 90 in Pulmonary Arterial Hypertension Treatment

Hsp90 levels and activities vary in different cell lines during the development of PAH. It was shown that the level and activity of Hsp90 were increased in PASMCs, leading to abnormal proliferation and migration of PASMCs and, thus, vascular remodeling eventually (Wang et al., 2016; Boucherat et al., 2018). In PAECs, the intracellular ATP level decreased due to mitochondrial dysfunction. This results in reduced activity of Hsp90, which in turn leads to the uncoupling of the interaction between eNOS and Hsp90, and decreased eNOS activity and NO production, resulting in further pulmonary vasoconstriction (García-Cardeña et al., 1998; Sud et al., 2008; Boucherat et al., 2018). The combined physiological changes increase PAP and pulmonary vascular resistance, aggravating the condition of PAH patients.

**FIGURE 5:**
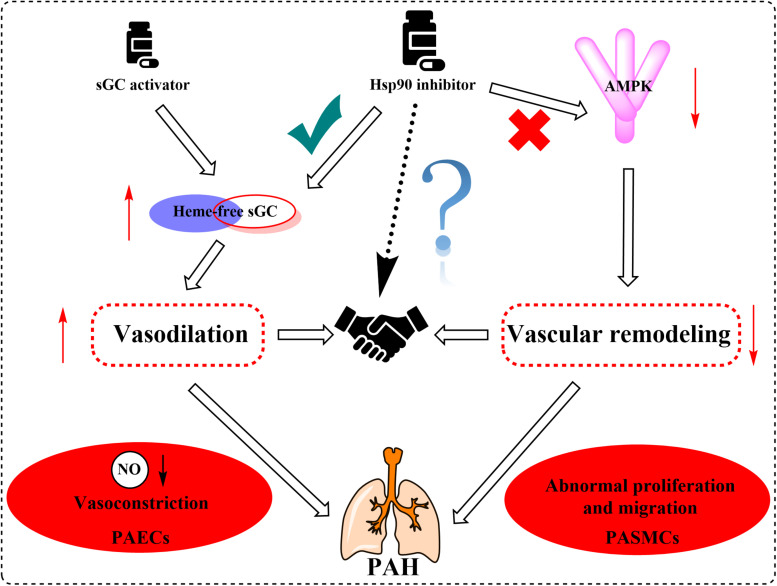
Proposed mechanism graph shows that the combination of Hsp90 inhibitors and sGC activators may lead to better treatment of PAH via relaxing blood vessels and inhibiting vascular remodeling.

Supported by long-term efficacy data and the latest European Society of Cardiology/European Respiratory Society guidelines, combination therapy is now regarded as the standard of care in PAH management and is becoming used widely in clinical practice (Galie et al., 2016; Lajoie et al., 2016; Burks et al., 2018). In this review, we summarized the multifaceted roles of Hsp90, such as the effects on sGC and AMPK, in PAH treatment. Based on the current situation and our new findings on the role of Hsp90 in PAH development, we speculate that the combination of Hsp90 inhibitors and sGC activators may provide significant benefit to PAH patients clinically (Figure 5).

## Conclusion

In summary, an increasing body of evidence has supported Hsp90 as a potential pathogenic factor in the development of PAH. It is known that PAH is a highly complicated and progressive disease, and no single drug has been consistently demonstrated to be efficient for patients with PAH. Combination therapy has shown potential advantages in long-term outcomes and achievements of predefined treatment goals. Therefore, regulators with vasodilation effect via sGC activation and vascular remodeling regulation effect by Hsp90 inhibition will provide more advantages and potentialities. Further elucidating the mechanisms of Hsp90 related pathways in the development of PAH will be essential for developing novel specific and safe therapies for this devastating disease.

## Author Contributions

LH and RZ wrote the manuscript. QBL and QLL contributed in revision. All authors read and approved the final manuscript.

## Conflict of Interest

The authors declare that the research was conducted in the absence of any commercial or financial relationships that could be construed as a potential conflict of interest.

## References

[B1] Ali M. M. Roe S. M. Vaughan C. K. Meyer P. Panaretou B. Piper P. W. (2006). Crystal structure of an Hsp90-nucleotide-p23/Sba1 closed chaperone complex. *Nature* 440 1013–1017. 10.1038/nature04716 16625188 PMC5703407

[B2] Balligand J. L. (2002). Heat shock protein 90 in endothelial nitric oxide synthase signaling: following the Lead(er)? *Circ Res.* 90 838–841. 10.1161/01.res.0000018173.10175.ff 11988482

[B3] Benza R. L. Miller D. P. Gomberg-Maitland M. Frantz R. P. Foreman A. J. Coffey C. S. (2010). Predicting survival in pulmonary arterial hypertension: insights from the Registry to evaluate early and long-term pulmonary arterial hypertension disease management (REVEAL). *Circulation* 122 164–172. 10.1161/CIRCULATIONAHA.109.898122 20585012

[B4] Boczek E. E. Reefschlager L. G. Dehling M. Struller T. J. Hausler E. Seidl A. (2015). Conformational processing of oncogenic v-Src kinase by the molecular chaperone Hsp90. *Proc. Natl. Acad. Sci. U.S.A.* 112 E3189–E3198. 10.1073/pnas.1424342112 26056257 PMC4485149

[B5] Boucherat O. Peterlini T. Bourgeois A. Nadeau V. Breuils-Bonnet S. Boilet-Molez S. (2018). Mitochondrial Hsp90 accumulation promotes vascular remodeling in pulmonary arterial hypertension. *Am. J. Respir. Crit. Care Med.* 198 90–103. 10.1164/rccm.201708-1751OC 29394093 PMC6835039

[B6] Boucherat O. Vitry G. Trinh I. Paulin R. Provencher S. Bonnet S. (2017). The cancer theory of pulmonary arterial hypertension. *Pulm. Circ.* 7 285–299. 10.1177/2045893217701438 28597757 PMC5467931

[B7] Budhiraja R. Tuder R. M. Hassoun P. M. (2004). Endothelial dysfunction in pulmonary hypertension. *Circulation* 109 159–165. 10.1161/01.CIR.0000102381.57477.50 14734504

[B8] Burks M. Stickel S. Galie N. (2018). Pulmonary arterial hypertension: combination therapy in practice. *Am. J. Cardiovasc. Drugs* 18 249–257. 10.1007/s40256-018-0272-5 29511993 PMC6028878

[B9] Chen W. Xiao H. Rizzo A. N. Zhang W. Mai Y. Ye M. (2014). Endothelial nitric oxide synthase dimerization is regulated by heat shock protein 90 rather than by phosphorylation. *PLoS One* 9:e105479. 10.1371/journal.pone.0105479 25153129 PMC4143281

[B10] Chen Y. Jiang B. Zhuang Y. Peng H. Chen W. (2017). Differential effects of heat shock protein 90 and serine 1179 phosphorylation on endothelial nitric oxide synthase activity and on its cofactors. *PLoS One* 12:e0179978. 10.1371/journal.pone.0179978 28654706 PMC5487052

[B11] Chen Z. Peng I. C. Sun W. Su M. I. Hsu P. H. Fu Y. (2009). AMP-activated protein kinase functionally phosphorylates endothelial nitric oxide synthase Ser633. *Circ. Res.* 104 496–505. 10.1161/CIRCRESAHA.108.187567 19131647 PMC2761102

[B12] Childers K. C. Garcin E. D. (2018). Structure/function of the soluble guanylyl cyclase catalytic domain. *Nitric Oxide* 77 53–64. 10.1016/j.niox.2018.04.008 29702251 PMC6005667

[B13] Dai J. Zhou Q. Chen J. Rexius-Hall M. L. Rehman J. Zhou G. (2018). Alpha-enolase regulates the malignant phenotype of pulmonary artery smooth muscle cells via the AMPK-Akt pathway. *Nat. Commun.* 9:3850. 10.1038/s41467-018-06376-x 30242159 PMC6155017

[B14] Dai Z. Zhu M. M. Peng Y. Machireddy N. Evans C. E. Machado R. (2018). Therapeutic targeting of vascular remodeling and right heart failure in pulmonary arterial hypertension with a HIF-2 alpha inhibitor. *Am. J. Respir. Crit. Care Med.* 198 1423–1434. 10.1164/rccm.201710-2079OC 29924941 PMC6290950

[B15] Dai Y. Schlanger S. Haque M. M. Misra S. Stuehr D. J. (2019). Heat shock protein 90 regulates soluble guanylyl cyclase maturation by a dual mechanism. *J. Biol. Chem.* 294 12880–12891. 10.1074/jbc.RA119.009016 31311859 PMC6721946

[B16] Dai Z. Zhao Y. Y. (2019). BET in pulmonary arterial hypertension: exploration of BET Inhibitors to reverse vascular remodeling. *Am. J. Respir. Crit. Care Med.* 200 806–808. 10.1164/rccm.201904-0877ED 31112388 PMC6812451

[B17] Dasgupta A. Bowman L. D’Arsigny C. L. Archer S. L. (2015). Soluble guanylate cyclase: a new therapeutic target for pulmonary arterial hypertension and chronic thromboembolic pulmonary hypertension. *Clin. Pharmacol. Ther.* 97 88–102. 10.1002/cpt.10 25670386 PMC4325399

[B18] Davis B. J. Xie Z. Viollet B. Zou M. H. (2006). Activation of the AMP-activated kinase by antidiabetes drug metformin stimulates nitric oxide synthesis in vivo by promoting the association of heat shock protein 90 and endothelial nitric oxide synthase. *Diabetes* 55 496–505. 10.2337/diabetes.55.02.06.db05-1064 16443786

[B19] Den R. B. Lu B. (2012). Heat shock protein 90 inhibition: rationale and clinical potential. *Ther. Adv. Med. Oncol.* 4 211–218. 10.1177/1758834012445574 22754594 PMC3384095

[B20] Derbyshire E. R. Marletta M. A. (2012). Structure and regulation of soluble guanylate cyclase. *Annu. Rev. Biochem.* 81 533–559. 10.1146/annurev-biochem-050410-100030 22404633

[B21] Dunham-Snary K. J. Wu D. Sykes E. A. Thakrar A. Parlow L. R. G. Mewburn J. D. (2017). Hypoxic pulmonary vasoconstriction: from molecular mechanisms to medicine. *Chest* 151 181–192. 10.1016/j.chest.2016.09.001 27645688 PMC5310129

[B22] Echeverria P. C. Briand P. A. Picard D. (2016). A remodeled Hsp90 molecular chaperone ensemble with the novel cochaperone aarsd1 is required for muscle differentiation. *Mol. Cell Biol.* 36 1310–1321. 10.1128/MCB.01099-15 26884463 PMC4836269

[B23] Falsone S. F. Leptihn S. Osterauer A. Haslbeck M. Buchner J. (2004). Oncogenic mutations reduce the stability of SRC kinase. *J. Mol. Biol.* 344 281–291. 10.1016/j.jmb.2004.08.091 15504417

[B24] Fan M. Park A. Nephew K. P. (2005). CHIP (carboxyl terminus of Hsp70-interacting protein) promotes basal and geldanamycin-induced degradation of estrogen receptor-alpha. *Mol. Endocrinol.* 19 2901–2914. 10.1210/me.2005-0111 16037132

[B25] Finka A. Goloubinoff P. (2013). Proteomic data from human cell cultures refine mechanisms of chaperone-mediated protein homeostasis. *Cell Stress Chaperones* 18 591–605. 10.1007/s12192-013-0413-3 23430704 PMC3745260

[B26] Follmann M. Griebenow N. Hahn M. G. Hartung I. Mais F. J. Mittendorf J. (2013). The chemistry and biology of soluble guanylate cyclase stimulators and activators. *Angew Chem. Int. Ed. Engl.* 52 9442–9462. 10.1002/anie.201302588 23963798

[B27] Fuhrmann-Stroissnigg H. Ling Y. Y. Zhao J. McGowan S. J. Zhu Y. Brooks R. W. (2017). Identification of Hsp90 inhibitors as a novel class of senolytics. *Nat. Commun.* 8:422. 10.1038/s41467-017-00314-z 28871086 PMC5583353

[B28] Galie N. Humbert M. Vachiery J. L. Gibbs S. Lang I. Torbicki A. (2016). 2015 ESC/ERS Guidelines for the diagnosis and treatment of pulmonary hypertension: the joint task force for the diagnosis and treatment of pulmonary hypertension of the european society of cardiology (ESC) and the european respiratory society (ERS): endorsed by: association for european paediatric and congenital cardiology (AEPC), international society for heart and lung transplantation (ISHLT). *Eur. Heart J.* 37 67–119. 10.1093/eurheartj/ehv317 26320113

[B29] García-Cardeña G. Fan R. Shah V. Sorrentino R. Cirino G. Papapetropoulos A. (1998). Dynamic activation of endothelial nitric oxide synthase by Hsp90. *Nature* 392 821–824. 10.1038/33934 9580552

[B30] Genest O. Wickner S. Doyle S. M. (2019). Hsp90 and Hsp70 chaperones: collaborators in protein remodeling. *J. Biol. Chem.* 294 2109–2120. 10.1074/jbc.REV118.002806 30401745 PMC6369297

[B31] Ghofrani H. A. Grimminger F. (2009). Soluble guanylate cyclase stimulation: an emerging option in pulmonary hypertension therapy. *Eur. Respir. Rev.* 18 35–41. 10.1183/09059180.00011112 20956121

[B32] Ghosh A. Stasch J. P. Papapetropoulos A. Stuehr D. J. (2014). Nitric oxide and heat shock protein 90 activate soluble guanylate cyclase by driving rapid change in its subunit interactions and heme content. *J. Biol. Chem.* 289 15259–15271. 10.1074/jbc.M114.559393 24733395 PMC4140884

[B33] Ghosh A. Stuehr D. J. (2017). Regulation of sGC via Hsp90, cellular heme, sGC agonists, and no: new pathways and clinical perspectives. *Antioxid. Redox Signal.* 26 182–190. 10.1089/ars.2016.6690 26983679 PMC5278824

[B34] Goasduff T. Cederbaum A. I. (2000). CYP2E1 degradation by in vitro reconstituted systems: role of the molecular chaperone Hsp90. *Arch. Biochem. Biophys.* 379 321–330. 10.1006/abbi.2000.1870 10898951

[B35] Graf C. Stankiewicz M. Kramer G. Mayer M. P. (2009). Spatially and kinetically resolved changes in the conformational dynamics of the Hsp90 chaperone machine. *EMBO J.* 28 602–613. 10.1038/emboj.2008.306 19165152 PMC2657576

[B36] Hessling M. Richter K. Buchner J. (2009). Dissection of the ATP-induced conformational cycle of the molecular chaperone Hsp90. *Nat. Struct. Mol. Biol.* 16 287–293. 10.1038/nsmb.1565 19234467

[B37] Hoter A. El-Sabban M. E. Naim H. Y. (2018). The Hsp90 family: structure, regulation, function, and implications in health and disease. *Int. J. Mol. Sci.* 19:2560. 10.3390/ijms19092560 30158430 PMC6164434

[B38] Huai Q. Wang H. Liu Y. Kim H. Y. Toft D. Ke H. (2005). Structures of the N-terminal and middle domains of E. *coli Hsp*90 and conformation changes upon ADP binding. *Structure* 13 579–590. 10.1016/j.str.2004.12.018 15837196

[B39] Humbert M. Morrell N. W. Archer S. L. Stenmark K. R. MacLean M. R. Lang I. M. (2004). Cellular and molecular pathobiology of pulmonary arterial hypertension. *J. Am. Coll. Cardiol.* 43 13S–24S. 10.1016/j.jacc.2004.02.029 15194174

[B40] Ibe J. C. Zhou Q. Chen T. Tang H. Yuan J. X. Raj J. U. (2013). Adenosine monophosphate-activated protein kinase is required for pulmonary artery smooth muscle cell survival and the development of hypoxic pulmonary hypertension. *Am. J. Respir. Cell Mol. Biol.* 49 609–618. 10.1165/rcmb.2012-0446OC 23668615 PMC3824043

[B41] Javid B. MacAry P. A. Lehner P. J. (2007). Structure and function: heat shock proteins and adaptive immunity. *J. Immunol.* 179 2035–2040. 10.4049/jimmunol.179.4.2035 17675458

[B42] Kang B. H. Plescia J. Song H. Y. Meli M. Colombo G. Beebe K. (2009). Combinatorial drug design targeting multiple cancer signaling networks controlled by mitochondrial Hsp90. *J. Clin. Invest.* 119 454–464. 10.1172/JCI37613 19229106 PMC2648691

[B43] Kang Y. Liu R. Wu J. X. Chen L. (2019). Structural insights into the mechanism of human soluble guanylate cyclase. *Nature* 574 206–210. 10.1038/s41586-019-1584-6 31514202

[B44] Kazlauskas A. Sundstrom S. Poellinger L. Pongratz I. (2001). The hsp90 chaperone complex regulates intracellular localization of the dioxin receptor. *Mol. Cell Biol.* 21 2594–2607. 10.1128/MCB.21.7.2594-2607.2001 11259606 PMC86890

[B45] Kirschke E. Goswami D. Southworth D. Griffin P. R. Agard D. A. (2014). Glucocorticoid receptor function regulated by coordinated action of the Hsp90 and Hsp70 chaperone cycles. *Cell* 157 1685–1697. 10.1016/j.cell.2014.04.038 24949977 PMC4087167

[B46] Lajoie A. C. Lauzière G. Lega J.-C. Lacasse Y. Martin S. Simard S. (2016). Combination therapy versus monotherapy for pulmonary arterial hypertension: a meta-analysis. *Lancet Respir. Med.* 4 291–305. 10.1016/s2213-2600(16)00027-8 26935844

[B47] Li J. Buchner J. (2013). Structure, function and regulation of the hsp90 machinery. *Biomed. J.* 36 106–117. 10.4103/2319-4170.113230 23806880

[B48] Li J. Richter K. Reinstein J. Buchner J. (2013). Integration of the accelerator Aha1 in the Hsp90 co-chaperone cycle. *Nat. Struct. Mol. Biol.* 20 326–331. 10.1038/nsmb.2502 23396352

[B49] Li L. Wang L. You Q. D. Xu X. L. (2020). Heat shock protein 90 inhibitors: an update on achievements, challenges, and future directions. *J. Med. Chem.* 63 1798–1822. 10.1021/acs.jmedchem.9b00940 31663736

[B50] Liu J. Wang W. Wang L. Chen S. Tian B. Huang K. (2018). IL-33 initiates vascular remodelling in hypoxic pulmonary hypertension by up-regulating HIF-1alpha and VEGF expression in vascular endothelial cells. *EBioMedicine* 33 196–210. 10.1016/j.ebiom.2018.06.003 29921553 PMC6085568

[B51] McClellan A. J. Xia Y. Deutschbauer A. M. Davis R. W. Gerstein M. Frydman J. (2007). Diverse cellular functions of the Hsp90 molecular chaperone uncovered using systems approaches. *Cell* 131 121–135. 10.1016/j.cell.2007.07.036 17923092

[B52] Meyer P. Prodromou C. Hu B. Vaughan C. Roe S. M. Panaretou B. (2003). Structural and functional analysis of the middle segment of Hsp90: implications for ATP hydrolysis and client protein and cochaperone interactions. *Mol. Cell* 11 647–658. 10.1016/s1097-2765(03)00065-0 12667448

[B53] Meyer P. Prodromou C. Liao C. Hu B. Roe S. M. Vaughan C. K. (2004). Structural basis for recruitment of the ATPase activator Aha1 to the Hsp90 chaperone machinery. *EMBO J.* 23 1402–1410. 10.1038/sj.emboj.7600141 15039704 PMC381413

[B54] Mickler M. Hessling M. Ratzke C. Buchner J. Hugel T. (2009). The large conformational changes of Hsp90 are only weakly coupled to ATP hydrolysis. *Nat. Struct. Mol. Biol.* 16 281–286. 10.1038/nsmb.1557 19234469

[B55] Mollapour M. Neckers L. (2012). Post-translational modifications of Hsp90 and their contributions to chaperone regulation. *Biochim. Biophys. Acta* 1823 648–655. 10.1016/j.bbamcr.2011.07.018 21856339 PMC3226900

[B56] Muller P. Ceskova P. Vojtesek B. (2005). Hsp90 is essential for restoring cellular functions of temperature-sensitive p53 mutant protein but not for stabilization and activation of wild-type p53: implications for cancer therapy. *J. Biol. Chem.* 280 6682–6691. 10.1074/jbc.M412767200 15613472

[B57] Nedvetsky P. I. Meurer S. Opitz N. Nedvetskaya T. Y. Muller H. Schmidt H. H. (2008). Heat shock protein 90 regulates stabilization rather than activation of soluble guanylate cyclase. *FEBS Lett.* 582 327–331. 10.1016/j.febslet.2007.12.025 18155168

[B58] Nguyen M. T. Csermely P. Soti C. (2013). Hsp90 chaperones PPARgamma and regulates differentiation and survival of 3T3-L1 adipocytes. *Cell Death Differ.* 20 1654–1663. 10.1038/cdd.2013.129 24096869 PMC3824595

[B59] Papapetropoulos A. Zhou Z. Gerassimou C. Yetik G. Venema R. C. Roussos C. (2005). Interaction between the 90-kDa heat shock protein and soluble guanylyl cyclase: physiological significance and mapping of the domains mediating binding. *Mol. Pharmacol.* 68 1133–1141. 10.1124/mol.105.012682 16024662

[B60] Paulin R. Courboulin A. Meloche J. Mainguy V. Dumas de la Roque E. Saksouk N. (2011). Signal transducers and activators of transcription-3/pim1 axis plays a critical role in the pathogenesis of human pulmonary arterial hypertension. *Circulation* 123 1205–1215. 10.1161/CIRCULATIONAHA.110.963314 21382889 PMC3545712

[B61] Pullen L. Bolon D. N. (2011). Enforced N-domain proximity stimulates Hsp90 ATPase activity and is compatible with function in vivo. *J. Biol. Chem.* 286 11091–11098. 10.1074/jbc.M111.223131 21278257 PMC3064163

[B62] Rabinovitch M. (2012). Molecular pathogenesis of pulmonary arterial hypertension. *J. Clin. Invest.* 122 4306–4313. 10.1172/JCI60658 23202738 PMC3533531

[B63] Ratzke C. Mickler M. Hellenkamp B. Buchner J. Hugel T. (2010). Dynamics of heat shock protein 90 C-terminal dimerization is an important part of its conformational cycle. *Proc. Natl. Acad. Sci. U.S.A.* 107 16101–16106. 10.1073/pnas.1000916107 20736353 PMC2941327

[B64] Richardson P. G. Mitsiades C. S. Laubach J. P. Lonial S. Chanan-Khan A. A. Anderson K. C. (2011). Inhibition of heat shock protein 90 (HSP90) as a therapeutic strategy for the treatment of myeloma and other cancers. *Br. J. Haematol.* 152 367–379. 10.1111/j.1365-2141.2010.08360.x 21219297

[B65] Ross F. A. MacKintosh C. Hardie D. G. (2016). AMP-activated protein kinase: a cellular energy sensor that comes in 12 flavours. *FEBS J.* 283 2987–3001. 10.1111/febs.13698 26934201 PMC4995730

[B66] Sakao S. Tatsumi K. (2011). Vascular remodeling in pulmonary arterial hypertension: multiple cancer-like pathways and possible treatment modalities. *Int. J. Cardiol.* 147 4–12. 10.1016/j.ijcard.2010.07.003 20692712

[B67] Sato S. Fujita N. Tsuruo T. (2000). Modulation of Akt kinase activity by binding to Hsp90. *Proc. Natl. Acad. Sci. U.S.A.* 97 10832–10837. 10.1073/pnas.170276797 10995457 PMC27109

[B68] Schermuly R. T. Stasch J. P. Pullamsetti S. S. Middendorff R. Muller D. Schluter K. D. (2008). Expression and function of soluble guanylate cyclase in pulmonary arterial hypertension. *Eur. Respir. J.* 32 881–891. 10.1183/09031936.00114407 18550612

[B69] Scheufler C. Brinker A. Bourenkov G. Pegoraro S. Moroder L. Bartunik H. (2000). Structure of TPR domain–peptide complexes. *Cell* 101 199–210. 10.1016/s0092-8674(00)80830-2 10786835

[B70] Schopf F. H. Biebl M. M. Buchner J. (2017). The HSP90 chaperone machinery. *Nat. Rev. Mol. Cell Biol.* 18 345–360. 10.1038/nrm.2017.20 28429788

[B71] Schulz E. Anter E. Zou M. H. Keaney J. F. Jr. (2005). Estradiol-mediated endothelial nitric oxide synthase association with heat shock protein 90 requires adenosine monophosphate-dependent protein kinase. *Circulation* 111 3473–3480. 10.1161/circulationaha.105.546812 15967841

[B72] Shah R. C. Sanker S. Wood K. C. Durgin B. G. Straub A. C. (2018). Redox regulation of soluble guanylyl cyclase. *Nitric Oxide* 76 97–104. 10.1016/j.niox.2018.03.013 29578056 PMC5916318

[B73] Shi W. Zhai C. Feng W. Wang J. Zhu Y. Li S. (2018). Resveratrol inhibits monocrotaline-induced pulmonary arterial remodeling by suppression of SphK1-mediated NF-kappaB activation. *Life Sci.* 210 140–149. 10.1016/j.lfs.2018.08.071 30179628

[B74] Shiau A. K. Harris S. F. Southworth D. R. Agard D. A. (2006). Structural analysis of E. *coli hsp*90 reveals dramatic nucleotide-dependent conformational rearrangements. *Cell* 127 329–340. 10.1016/j.cell.2006.09.027 17055434

[B75] Shimoda L. A. Laurie S. S. (2013). Vascular remodeling in pulmonary hypertension. *J. Mol. Med.* 91 297–309. 10.1007/s00109-013-0998-0 23334338 PMC3584237

[B76] Simonneau G. Montani D. Celermajer D. S. Denton C. P. Gatzoulis M. A. Krowka M. (2019). Haemodynamic definitions and updated clinical classification of pulmonary hypertension. *Eur. Respir. J.* 53:1801913. 10.1183/13993003.01913-2018 30545968 PMC6351336

[B77] Sreedhar A. S. Kalmár E. Csermely P. Shen Y. F. (2004). Hsp90 isoforms: functions, expression and clinical importance. *FEBS Lett.* 562 11–15. 10.1016/s0014-5793(04)00229-7 15069952

[B78] Stasch J. P. Pacher P. Evgenov O. V. (2011). Soluble guanylate cyclase as an emerging therapeutic target in cardiopulmonary disease. *Circulation* 123 2263–2273. 10.1161/CIRCULATIONAHA.110.981738 21606405 PMC3103045

[B79] Street T. O. Lavery L. A. Agard D. A. (2011). Substrate binding drives large-scale conformational changes in the Hsp90 molecular chaperone. *Mol. Cell* 42 96–105. 10.1016/j.molcel.2011.01.029 21474071 PMC3105473

[B80] Sud N. Wells S. M. Sharma S. Wiseman D. A. Wilham J. Black S. M. (2008). Asymmetric dimethylarginine inhibits Hsp90 activity in pulmonary arterial endothelial cells: role of mitochondrial dysfunction. *Am. J. Physiol. Cell Physiol.* 294 C1407–C1418. 10.1152/ajpcell.00384.2007 18385287 PMC3815615

[B81] Taipale M. Jarosz D. F. Lindquist S. (2010). Hsp90 at the hub of protein homeostasis: emerging mechanistic insights. *Nat. Rev. Mol. Cell Biol.* 11 515–528. 10.1038/nrm2918 20531426

[B82] Thenappan T. Ormiston M. L. Ryan J. J. Archer S. L. (2018). Pulmonary arterial hypertension: pathogenesis and clinical management. *BMJ* 360:j5492. 10.1136/bmj.j5492 29540357 PMC6889979

[B83] Thenappan T. Shah S. J. Rich S. Tian L. Archer S. L. Gomberg-Maitland M. (2010). Survival in pulmonary arterial hypertension: a reappraisal of the NIH risk stratification equation. *Eur. Respir. J.* 35 1079–1087. 10.1183/09031936.00072709 20032020 PMC8782564

[B84] Theodoraki M. A. Caplan A. J. (2012). Quality control and fate determination of Hsp90 client proteins. *Biochim. Biophys. Acta* 1823 683–688. 10.1016/j.bbamcr.2011.08.006 21871502 PMC3242914

[B85] Thompson A. A. R. Lawrie A. (2017). Targeting vascular remodeling to treat pulmonary arterial hypertension. *Trends Mol. Med.* 23 31–45. 10.1016/j.molmed.2016.11.005 27989641

[B86] Tsutsumi S. Mollapour M. Graf C. Lee C. T. Scroggins B. T. Xu W. (2009). Hsp90 charged-linker truncation reverses the functional consequences of weakened hydrophobic contacts in the N domain. *Nat. Struct. Mol. Biol.* 16 1141–1147. 10.1038/nsmb.1682 19838189 PMC8459299

[B87] Tuder R. M. Archer S. L. Dorfmuller P. Erzurum S. C. Guignabert C. Michelakis E. (2013). Relevant issues in the pathology and pathobiology of pulmonary hypertension. *J. Am. Coll. Cardiol.* 62 D4–D12. 10.1016/j.jacc.2013.10.025 24355640 PMC3970402

[B88] Wang G. K. Li S. H. Zhao Z. M. Liu S. X. Zhang G. X. Yang F. (2016). Inhibition of heat shock protein 90 improves pulmonary arteriole remodeling in pulmonary arterial hypertension. *Oncotarget* 7 54263–55427. 10.18632/oncotarget.10855 27472464 PMC5342340

[B89] Willows R. Sanders M. J. Xiao B. Patel B. R. Martin S. R. Read J. (2017). Phosphorylation of AMPK by upstream kinases is required for activity in mammalian cells. *Biochem. J.* 474 3059–3073. 10.1042/BCJ20170458 28694351 PMC5565919

[B90] Wu J. Liu T. Rios Z. Mei Q. Lin X. Cao S. (2017). Heat shock proteins and cancer. *Trends Pharmacol. Sci.* 38 226–256. 10.1016/j.tips.2016.11.009 28012700

[B91] Xia T. Dimitropoulou C. Zeng J. Antonova G. N. Snead C. Venema R. C. (2007). Chaperone-dependent E3 ligase CHIP ubiquitinates and mediates proteasomal degradation of soluble guanylyl cyclase. *Am. J. Physiol. Heart Circ. Physiol.* 293 H3080–H3087. 10.1152/ajpheart.00579.2007 17873020

[B92] Xiao Y. Liu Y. (2020). Recent advances in the discovery of novel Hsp90 inhibitors: an update from 2014. *Curr. Drug Targets* 21 302–317. 10.2174/1389450120666190829162544 31465284

[B93] Yetik-Anacak G. Xia T. Dimitropoulou C. Venema R. C. Catravas J. D. (2006). Effects of Hsp90 binding inhibitors on sGC-mediated vascular relaxation. *Am. J. Physiol. Heart Circ. Physiol.* 291 H260–H268. 10.1152/ajpheart.01027.2005 16489110

[B94] Young J. C. Agashe V. R. Siegers K. Hartl F. U. (2004). Pathways of chaperone-mediated protein folding in the cytosol. *Nat. Rev. Mol. Cell Biol.* 5 781–791. 10.1038/nrm1492 15459659

[B95] Zhang L. Yi Y. Guo Q. Sun Y. Ma S. Xiao S. (2012). Hsp90 interacts with AMPK and mediates acetyl-CoA carboxylase phosphorylation. *Cell Signal.* 24 859–865. 10.1016/j.cellsig.2011.12.001 22178220

[B96] Zhao R. Davey M. Hsu Y. C. Kaplanek P. Tong A. Parsons A. B. (2005). Navigating the chaperone network: an integrative map of physical and genetic interactions mediated by the Hsp90 chaperone. *Cell* 120 715–727. 10.1016/j.cell.2004.12.024 15766533

[B97] Zuehlke A. D. Moses M. A. Neckers L. (2018). Heat shock protein 90: its inhibition and function. *Philos. Trans. R. Soc. Lond B Biol. Sci.* 373:20160527. 10.1098/rstb.2016.0527 29203712 PMC5717527

